# Data extraction from machine-translated versus original language randomized trial reports: a comparative study

**DOI:** 10.1186/2046-4053-2-97

**Published:** 2013-11-07

**Authors:** Ethan M Balk, Mei Chung, Minghua L Chen, Lina Kong Win Chang, Thomas A Trikalinos

**Affiliations:** 1 Tufts Evidence-based Practice Center, Institute for Clinical Research and Health Policy Studies, 800 Washington Street, Box 63, Boston, MA 02111, USA; 2 Center for Evidence-based Medicine, Brown University, 121 South Main Street, 8th floor, Providence, RI 02912, USA

**Keywords:** Data extraction, Machine translation, Randomized controlled trials, Systematic review

## Abstract

**Background:**

Google Translate offers free Web-based translation, but it is unknown whether its translation accuracy is sufficient to use in systematic reviews to mitigate concerns about language bias.

**Methods:**

We compared data extraction from non-English language studies with extraction from translations by Google Translate of 10 studies in each of five languages (Chinese, French, German, Japanese and Spanish). Fluent speakers double-extracted original-language articles. Researchers who did not speak the given language double-extracted translated articles along with 10 additional English language trials. Using the original language extractions as a gold standard, we estimated the probability and odds ratio of correctly extracting items from translated articles compared with English, adjusting for reviewer and language.

**Results:**

Translation required about 30 minutes per article and extraction of translated articles required additional extraction time. The likelihood of correct extractions was greater for study design and intervention domain items than for outcome descriptions and, particularly, study results. Translated Spanish articles yielded the highest percentage of items (93%) that were correctly extracted more than half the time (followed by German and Japanese 89%, French 85%, and Chinese 78%) but Chinese articles yielded the highest percentage of items (41%) that were correctly extracted >98% of the time (followed by Spanish 30%, French 26%, German 22%, and Japanese 19%). In general, extractors’ confidence in translations was not associated with their accuracy.

**Conclusions:**

Translation by Google Translate generally required few resources. Based on our analysis of translations from five languages, using machine translation has the potential to reduce language bias in systematic reviews; however, pending additional empirical data, reviewers should be cautious about using translated data. There remains a trade-off between completeness of systematic reviews (including all available studies) and risk of error (due to poor translation).

## Background

Systematic reviews commonly restrict literature searches to English language publications, as was done in 28 of 38 recent US-based Evidence-based Practice Center reports [1]. The most commonly reported reason for language restriction was a lack of resources or prohibitive translation costs. This approach may result in selection bias based on language [2] and may not follow the Institute of Medicine’s standards for conducting systematic reviews [3]. However, formally translating articles is costly and resource-intensive. Therefore, a reliable, low-cost, easily available service to translate articles may allow investigators to easily broaden the scope of their systematic reviews and diminish possible language bias.

Google Translate® is a free, Web-based program with a reputation for accurate, natural translation [4]. Whether the translations are adequate for accurate systematic review data extraction is unclear. A pilot study that used Google Translate on 11 German articles found that inter-rater agreement was 73% (κ = 0.38) for whether the article should be included in the review [5]. But no study has tested whether the translations are sufficiently accurate for use in systematic review.

We wanted to evaluate the accuracy of the freely available, online translation tool Google Translate for the purposes of data extraction of articles in selected non-English languages. We aimed to compare data extraction of trials done on original-language articles by native speakers with data extraction done on articles translated to English by Google Translate. We also tracked and enumerated the time and resources used for article translation and the extra effort required for data extraction related to use of translated articles.

## Methods

More complete details of our methods are described in the Research Methods Report for the Agency for Health Research and Quality [6].

### Study selection

We included articles in five languages: Chinese, French, German, Japanese and Spanish. We searched MEDLINE with the term ‘randomized controlled trial’, restricted to each language. Working in reverse chronological order, we accepted the first 10 publications we found in each language, regardless of topic, for which either a machine-readable pdf or html file was available for the full text of the article (that we could translate with Google Translate). We also chose 10 English-language trials that were published in a distribution of years roughly corresponding to the distribution of the non-English articles. The list of included studies is presented in Additional file 1 and their characteristics in Additional file 2: Table S1. With this number of studies, the observed power to detect differences between extractions of translated and untranslated articles was above 80%.

### Translation process

We translated each article into English using Google Translate. We used the simplest method possible for each article, including one-step translation of complete articles available as Web pages (html) or as pdf files; copying and pasting blocks of text from pdf files into Google Translate; or copying text into word processing software, reformatting the text, and then copying into Google Translate. We included the English translations, any English-language abstracts that were published with the original articles, and images of figures and tables that could not be translated due to formatting issues. Translations were performed primarily by a research assistant who estimated the approximate time she required to translate each article.

### Data extraction process

A description of the data extractors and a flowchart of the basic processes employed for extracting, reconciling and analyzing articles are provided in Additional files 3 and 4. Each original language version of the articles was double extracted by two fluent readers. The extractors were informed of disagreements and asked to recheck discrepancies. The extractions were then reconciled allowing multiple ‘correct’ answers if the extractors interpreted the data differently. This approach was taken to reduce the likelihood of disagreements between native and translated extractions resulting from differences in interpretation rather than disagreements due to poor translation. The reconciled extractions from the fluent readers were considered to be the reference standard extractions. The Google translated version of each article was extracted by two researchers who did not speak the article language, out of a pool of eight extractors. These eight researchers also extracted the 10 English language articles. Reconciliation of the extractions of English language articles was conducted by consensus either among five of eight extractors or, failing that, agreement between the two senior researchers (EMB, TAT), again allowing for multiple ‘correct’ answers if data appeared to have been interpreted differently.

### Data extraction form and comparison

We focused data extraction on common and important study domains for systematic review: study design and methods, interventions (and comparators), outcomes, and results. Extractors provided a rough estimate of how much extra time they spent with the article compared with the time they would have spent extracting a similar English article. They also reported their level of confidence in the accuracy and translation of the article. Additional file 5 lists the data extraction items.

When possible, we selected one categorical and one continuous outcome from each trial. We limited the extraction of results to two interventions. Prior to data extraction, for each language we compiled a list of about a dozen outcomes that were reported in at least one article in that language. We aimed for a mix of primary and secondary outcomes, and clinical and intermediate or surrogate outcomes. Researchers were asked to check off all outcomes from the lists that were reported in the article.

For the comparisons of translations and of English extractions with their reference standards, each data item was coded as agree or disagree. ‘Disagree’ included erroneous data, incomplete data, and data items incorrectly extracted as not reported.

### Analysis

We used a generalized linear mixed-effects model to examine whether the probability of correctly extracting the item was related to the language of the original publication and to each extractor’s likelihood of correctly extracting English articles, accounting for the fact that extractions were grouped by paper. The model used the pattern of allocation of extractors to languages to control for reviewer effects. For each item, we report the model-predicted percent accuracy for an ‘average reviewer’ and the odds ratios for correct extraction of translated articles compared with correct extraction of English-language papers. We constructed an average reviewer by using the mean of the reviewer-specific coefficients to obtain model predictions. When the model did not converge, we ignored reviewer effects and calculated ‘crude’ percentages and odds ratios. We analyzed cases when the odds of correctly extracting individual items from the translated articles was greater than the odds of doing so from the extracted English articles (when the odds ratio was >1) as equivalent to perfect agreement.

### Role of the funding source

This work was funded under contract from the US Agency for Health Research and Quality, US Department of Health and Human Services, which did not participate in data analysis or preparation, review, or approval of the manuscript for publication. The funder did not participate in the conception, design, conduct, analysis or decision to submit this manuscript for publication.

## Results and discussion

### Article translation

The length of time our research assistant required to translate articles ranged from 5 minutes (2 of 50 articles) to about 1 hour (11 articles) for most articles; two articles took >1 hour. Excluding the time taken for the latter two articles, the median time to translate was about 30 minutes, though Chinese articles generally required 1 hour (Additional file 2: Table S2).

### Data extraction from translated articles

The researchers who extracted data from translated articles estimated that their extractions likely took more time than extraction from equivalent English language articles would have taken. For 56% of Spanish articles, extractors estimated they took <5 additional minutes to extract data, and all but one article took <30 additional minutes. Between 60% and 75% of other language articles were estimated to take between 6 and 30 additional minutes for data extraction (Additional file 2: Table S3).

### Comparison of extractions from translated and original articles

Additional file 6 displays the adjusted estimated percentage of correct extractions per language, including English, and per analyzed extraction item. In general, the agreement across languages between the extractors and the reference standards for each article was greater for items in design and intervention domains than for items in outcome description and study result domains. In particular, extractors did relatively poorly extracting which outcomes from a given list were reported in the study and in extracting net differences and their standard errors for continuous outcomes. Translated Chinese articles yielded the largest percentage of items (22%) that were incorrectly extracted by more than half the extractors, although Chinese articles also yielded the largest percentage of items (41%) that were each extracted correctly by >98% of the extractors (including English article extractions). Translated Chinese articles had lower likelihoods of correct extractions for the items in the important intervention description, outcome description and results domains. By contrast, extractors of translated Spanish articles had relatively high likelihoods of extracting items correctly except, in comparison with English, for results data. For Spanish, only 7% of items had <50% correct extractions, including funding source and identifying reported outcomes, but only 30% of items were extracted correctly by >98% of the extractors. Extractions of other translated language articles yielded similar patterns as for translated Chinese articles, but with generally higher rates of correct extractions.

Additional file 7 displays the adjusted odds ratios between translated and English articles of correctly extracting individual items. Overall, the pattern of adjusted odds ratios of correct answers compared with English across items and languages was similar to the pattern of adjusted percentages. It highlights that, for all translated languages except Spanish, extractors were statistically significantly more likely to extract incorrect data for outcome description and results from translated articles than from English articles. Similarly, the likelihood of missing reported outcomes was higher from translated articles, significantly so for German, Japanese and Spanish articles.

### Association between extractor confidence and accuracy

Extractors had strong confidence in the translations for the majority (60%) of Spanish articles (Additional file 2: Table S4). Confidence in the translation of other language articles was mostly moderate (60% to 65%). For French articles, the accuracy was considerably higher when extractors had strong confidence (94% accuracy across articles and items) than moderate or little confidence (67% accuracy). However, this pattern was not seen for other languages and, of note, the accuracy for Chinese articles was higher when extractors had little confidence (88%) than when they had moderate or strong confidence (73%). Overall, across all languages, accuracy was about the same regardless of extractors’ confidence level (76% to 79%).

## Conclusions

Our results demonstrated that using Google Translate to translate medical articles may be feasible, is not resource-intensive, and leads to operationally workable English versions. However, accuracy may not be considered to be sufficiently high to meet the standards for many systematic reviews. The accuracy of translation was dependent on the original language of the article. Specifically, extractions of Spanish articles were most accurate, followed by somewhat less accurate extractions from German, Japanese and French articles. The least accurate data extractions resulted from translated Chinese articles. We found good levels of agreement (mostly >85%) for extraction of most study design questions and generally good levels of agreement (mostly >70%) for extraction of descriptions of the intervention and the number of participants randomized. The accuracy of descriptions of outcomes and results data varied widely by language, with descriptions of outcomes being commonly inaccurate from Chinese, German and French articles; continuous results data being inaccurate from French and Chinese articles; categorical results data being inaccurate from German and Japanese articles; and *P*-values being inaccurate in Japanese and Chinese articles. Translated Spanish articles generally yielded more accurate outcome descriptions and results data. However, there are numerous reasons that accuracy may have varied across languages. Google Translate may be poorer at translating scientific articles in some languages than others (which appeared to be the case for Chinese), or the underlying quality and clarity of the writing may be systematically different for authors in different languages. But apparent differences may also have arisen from random variation in the relatively small sample of papers included in each language.

Although data extraction from translated articles was assessed to be more difficult and time consuming than extraction from equivalent English language articles, extraction was always feasible in what was considered to be a reasonable amount of time, even including the extra time required to perform article translation. We expected to find that investigators would provide more accurate extractions when they had greater confidence in the accuracy and completeness of the translations. However, with the possible exception of French studies, we did not find this to be the case. It is unclear why the data extractors were not more confident about studies they more accurately extracted. It may be that they were unable to disambiguate difficulties in extracting the studies due to poor translation from those due to poor reporting. This finding should not be over-interpreted but it does call into question whether extractors can subjectively assess how accurate their extractions from translated articles are.

Despite double data extraction of original language articles and adjustment for accuracy of extraction of English language articles, we did not fully remove the possibility that differences between languages were in part due to intrinsic differences between data extractors or the different articles in the different languages. In particular, we could not fully control for the likelihood that extractors made errors unrelated to translation in specific articles. Disagreements between reviewers and data extraction errors are not uncommon even in systematic reviews of English language articles. Re-analyses of published meta-analyses and studies assessing extraction error rates have found discrepancies with the original papers in about one quarter to two thirds [7]–[10].

Differences in extraction accuracy may also have resulted from fundamental differences in the studies across different languages; in the medical fields being examined; and in the complexity of the study designs, interventions, outcomes and analyses. Likely, the data extraction error rate was higher than for a typical systematic review because the articles were on random topics and the data extractors were neither trained nor necessarily proficient in the clinical domains. Therefore, our conclusions may provide a more pessimistic view of the performance of Google Translate than would be the case in actual systematic reviews, where greater familiarity with the topic and related studies would likely improve accuracy of interpreting and extracting translated articles.

We did not have extractors of translated articles reconcile their extractions and then compare the reconciled translated and reconciled original language extractions. Doing so might have more closely mimicked typical systematic review methods, but would have greatly reduced the study’s power. However, despite our power calculation, the confidence intervals of the adjusted odds ratios between translated and English articles were generally wide, possibly resulting in either an overestimation of the number of items with ‘trends’ toward large differences in accuracy (that is, small but non-significant odds ratios) or an underestimation of the number of true effects (due to frequent non-significance). An alternative strategy would have been to have native speakers first extract translated articles and then extract the same untranslated article. However, this may have introduced other methodological concerns related to loss of blinding. This study did not assess the impact of including machine-translated (or human translator-translated) studies on meta-analysis or systematic review results; instead it assessed the accuracy of a readily accessible machine translation tool.

A reasonable interpretation is that translation software is still sufficiently inaccurate for use in systematic review; that the risk of introducing errors is too great. However, we suggest that for many systematic reviews, investigators may find it worthwhile to include non-English language articles translated with Google Translate, at least in sensitivity analyses. Even though Google translation of medical articles in most cases is far from perfect and on average results in higher levels of inaccuracies than extraction from English, we suggest that the technique has potential to be of value. For most of the tested languages, it may be reasonable to attempt translation and extraction of non-English language articles that are available as machine-readable pdf (or html) files. A major caveat, though, is that the extraction of results data was least accurate. Thus, extra care should be taken when considering results data from translated articles. It would be appropriate to consistently perform sensitivity analyses regarding translated articles. Where differences in findings do occur when translated articles are included or omitted, it should be recognized that any differences may be due not only to differences in applicability or methodology, but to errors in translation. Each investigator considering the inclusion of articles requiring machine translation into a systematic review will need to decide the appropriate balance between completeness and risk of extraction errors.

We conclude that it is a reasonable option for systematic reviewers to devote the small amount of resources and effort necessary to try Google Translate to include non-English articles. It will be important, however, to recognize that extraction of these articles is more prone to error than extraction of typical English language articles. Therefore, judgment will be needed to determine how much confidence the reviewers have in the accuracy of the data extraction of these articles, and to recognize that apparently missing or unclearly reported data may be more a factor of poor translation than of poor methodology. However, given the limited evidence regarding the accuracy of machine translation, which suggests a higher error rate than extraction from English articles, it would be equally reasonable for investigators to forgo machine translation and inclusion of non-English language articles.

## Competing interests

The authors declare that they have no competing interests.

## Authors’ contributions

EMB, MC and TAT made substantial contributions to the study conception and design. EMB, MC, MLC, LKWC and TAT participated in acquisition of data. EMB and TAT were principally responsible for analysis and interpretation of data. EMB, MC, MLC, LKWC and TAT drafted or critically revised the manuscript for important intellectual content. All authors read and approved the final manuscript.

## Supplementary Material

Additional file 1 List of included and translated articles. Click here for file

Additional file 2: Table S1 Characteristics of included trials. **Table S2**: Translation time (minutes), by language. **Table S3**: Estimated additional time required compared to extraction of a similar English article. **Table S4**: Confidence in accuracy and completeness of the translation. Click here for file

Additional file 3 Summary description of data extractors. Click here for file

Additional file 4 Flowchart of basic processes. Click here for file

Additional file 5 Data extraction items. Click here for file

Additional file 6 Percentage of correct extractions, per item and language, adjusted for individual’s likelihood of correctly extracting the same data item from English articles. Click here for file

Additional file 7 **Odds ratios of correct extractions, compared with English, adjusted for individual’s likelihood of correctly extracting the same data item from English articles.** The extraction items are sorted to match Additional file 6. Shading of cells matches is based on the odds ratio and statistical significance. Darker shading indicates greater inaccuracy; NS = non-significant; OR = odds ratio. Click here for file
