# Supplementary material for: Data extraction from machine-translated versus original language randomized trial reports: a comparative study
Source: Syst Rev. 2013 Nov 7;2:97. doi: 10.1186/2046-4053-2-97 (PMC4226266; doi:10.1186/2046-4053-2-97)
Supplement: Additional file 1 — List of included and translated articles. [file 2046-4053-2-97-S1.docx]

Additional file 1. List of included and translated articles

### Chinese

Hu XY, Zhou YX, Xu SZ, Lin YY. [Effects of probiotics on feeding intolerance in low birth weight premature infants]. [Chinese]. *Zhongguo Dang Dai Er Ke Za Zhi.* 2010;12:693-695. PMID 20849715.

Li H, Dong L, Li Y, Fu S. [A randomized clinical trial of combination of Aidi injection with Gemcitabine and Oxaliplatin regimen or Go regimen only in the treatment of advanced non-small-cell lung cancer.]. [Chinese]. *Zhongguo Fei Ai Za Zhi.* 2008;11:570-573. PMID 20735973.

Liu X, Liu D, Li J, Ou D, Zhou Z. [Safety and efficacy of carbon dioxide insufflation during colonoscopy]. [Chinese]. *Zhong Nan Da Xue Xue Bao Yi Xue Ban.* 2009;34:825-829. PMID 19734597.

Tang FZ, Liu YL, Wen FQ, Zhang ZX. [Comparison of therapeutic effects in severe nocturia: gradual versus immediate drug withdrawal]. [Chinese]. *Zhongguo Dang Dai Er Ke Za Zhi.* 2010;12:198-200. PMID 20350430.

Wang P, Yang J, Liu G, Chen H, Yang F. [Effects of moxibustion at head-points on levels of somatostatin and arginine vasopressin from cerebrospinal fluid in patients with vascular dementia: a randomized controlled trial]. [Chinese]. *Zhong Xi Yi Jie He Xue Bao.* 2010;8:636-640. PMID 20619139.

Xu JS, Yang JW, Gu MN, Chen YM. [Effects of fentanyl on EC50 of ropivacaine for postoperative epidural analgesia after gynecological surgery]. [Chinese]. *Di Yi Jun Yi Da Xue Xue Bao.* 2004;24:1326-1327. PMID 15567796.

Xu XH, Chang YT, Li L, Li J, Zhang DM, Zou XH. [Effect of fructose-1,6-diphosphete on myocardial preservation during pulmonary operations]. [Chinese]. *Zhong Nan Da Xue Xue Bao Yi Xue Ban.* 2008;33:966-969. PMID 19001742.

Yang MH, Li M, Dou YQ et al. [Effects of Bushen Huoxue Granule on motor function in patients with Parkinson's disease: a multicenter, randomized, double-blind and placebo-controlled trial]. [Chinese]. *Zhong Xi Yi Jie He Xue Bao.* 2010;8:231-237. PMID 20226144.

Yi JH, Li RR. [Influence of near-work and outdoor activities on myopia progression in school children]. [Chinese]. *Zhongguo Dang Dai Er Ke Za Zhi.* 2011;13:32-35. PMID 21251384.

Zhang GQ, Ge L, Ding W, Li HJ. [The value of portal vein chemotherapy after radical resection in delaying intrahepatic recurrence of stage II primary hepatocellular carcinoma]. [Chinese]. *Ai Zheng.* 2008;27:1297-1301. PMID 19079997.

### French

Aubin M, Vezina L, Maziade J, Robitaille NM. [Control of arterial hypertension: effectiveness of an intervention performed by family practitioners]. [French]. *Can Fam Physician.* 1994;40:1742-1752. PMID 7950469.

Aydin A, Karadayi K, Aykan U, Can G, Colakoglu K, Bilge AH. [Effectiveness of topical ciclosporin A treatment after excision of primary pterygium and limbal conjunctival autograft]. [French]. *J Fr Ophtalmol.* 2008;31:699-704. PMID 18971855.

Baillargeon L, Drouin J, Desjardins L, Leroux D, Audet D. [The effects of Arnica Montana on blood coagulation. Randomized controlled trial]. [French]. *Can Fam Physician.* 1993;39:2362-2367. PMID 7903572.

Devogelaere T, Beresniak A, Raymaeckers A, Naacke H, Ssi YK, I, Bremond-Gignac D. [Clinical study of Supranettes pads in the treatment of seasonal or perennial allergic conjunctivitis in children]. [French]. *J Fr Ophtalmol.* 2006;29:593-598. PMID 16885888.

Fekih M, Ben ZN, Jnifen A et al. [Comparing two Prepidil gel regimens for cervical ripening before induction of labor at term: a randomized trial]. [French]. *J Gynecol Obstet Biol Reprod (Paris).* 2009;38:335-340. PMID 19467806.

Gadioux-Madern F, Lelez ML, Sellami L et al. [Influence of the instillation of two versus three eyedrops of cyclopentolate 0.5% on refraction of Caucasian nonstrabismic children]. [French]. *J Fr Ophtalmol.* 2008;31:51-55. PMID 18401299.

Gosselin P, Verreault R, Gaudreault C, Guillemette J. [Dietary treatment of mild to moderate hypercholesterolemia. Effectiveness of different interventions]. [French]. *Can Fam Physician.* 1996;42:2160-2167. PMID 8974552.

Lamouliatte H, Perie F, Joubert-Collin M. [Treatment of Helicobacter pylori infection with lansoprazole 30 mg or 60 mg combined with two antibiotics for duodenal ulcers]. [French]. *Gastroenterol Clin Biol.* 2000;24:495-500. PMID 10891736.

Polonovski JM, El MM. [Treatment of acute maxillary sinusitis in adults. Comparison of cefpodoxime-proxetil and amoxicillin-clavulanic acid]. [French]. *Presse Med.* 2006;35:33-38. PMID 16462661.

Rolachon A, Kezachian G, Causse X et al. [Value of high-dose interferon-alpha in chronic viral hepatitis C patients non-responder to a 1st treatment. Pilot study prospective and randomized trial]. [French]. *Gastroenterol Clin Biol.* 1997;21:924-928. PMID 9587555.

### German

Bechdolf A, Pohlmann B, Guttgemanns J et al. [State-dependent motivational interviewing for people with schizophrenia and substance use : Results of a randomised controlled trial]. [German]. *Nervenarzt.* 2012;83:888-896. PMID 21720841.

Birnbaum F, Schwartzkopff J, Bohringer D, Reinhard T. [Penetrating keratoplasty with intrastromal corneal ring. A prospective randomized study]. [German]. *Ophthalmologe.* 2008;105:452-456. PMID 17899113.

Borner M, Burkle H, Trojan S, Horoshun G, Riewendt HD, Wappler F. [Intra-articular ketamine after arthroscopic knee surgery. Optimisation of postoperative analgesia]. [German]. *Anaesthesist.* 2007;56:1120-1127. PMID 17726586.

Langer C, Forster H, Konietschke F et al. [Mesh shrinkage in hernia surgery: data from a prospective randomized double-blinded clinical study]. [German]. *Chirurg.* 2010;81:735. PMID 20186380.

Marx S, Cimniak U, Beckert R, Schwerla F, Resch KL. [Chronic prostatitis/chronic pelvic pain syndrome. Influence of osteopathic treatment - a randomized controlled study]. [German]. *Urologe A.* 2009;48:1339-1345. PMID 19705093.

Meybohm P, Hanss R, Bein B et al. [Comparison of premedication regimes. A randomized, controlled trial]. [German]. *Anaesthesist.* 2007;56:890-896. PMID 17551699.

Schnabel M, Vassiliou T, Schmidt T et al. [Results of early mobilisation of acute whiplash injuries]. [German]. *Schmerz.* 2002;16:15-21. PMID 11845337.

Stoffels I, Wolter TP, Sailer AM, Pallua N. [The impact of silicone spray on scar formation. A single-center placebo-controlled double-blind trial]. [German]. *Hautarzt.* 2010;61:332-338. PMID 19967328.

Warlo I, Krummenauer F, Dick HB. [Rotational stability in intraocular lenses with C-loop haptics versus Z haptics in cataract surgery. A prospective randomised comparison]. [German]. *Ophthalmologe.* 2005;102:987-992. PMID 15785909.

Wohlrab D, Droege JW, Mendel T et al. [Minimally invasive vs. transgluteal total hip replacement. A 3-month follow-up of a prospective randomized clinical study]. [German]. *Orthopade.* 2008;37:1121-1126. PMID 18810386.

### Japanese

Adachi Y, Sumikuma T, Kagami R et al. [Improvement of patient adherence by mixing oral itraconazole solution with a beverage (orange juice)]. [Japanese]. *Rinsho Ketsueki.* 2010;51:315-319. PMID 20534951.

Hirata K, Nakahara S, Shimokobe T et al. [A randomized controlled trial of postoperative adjuvant chemotherapy for colorectal cancer-optimal duration of the treatment]. [Japanese]. *Gan To Kagaku Ryoho.* 2009;36:77-82. PMID 19151567.

Kurokawa M, Masuda Y, Noda M et al. [Minimal effective dose on serum cholesterol concentration and the safety evaluation of dressing containing plant sterol in Japanese subjects]. [Japanese]. *J Oleo Sci.* 2008;57:23-33. PMID 18075220.

Miura H, Takahashi Y, Kitabatake Y. [Influence of group training on pulse wave velocity in elderly women]. [Japanese]. *Nihon Koshu Eisei Zasshi.* 2010;57:271-278. PMID 20560409.

Mochizuki M, Hatsugaya M, Rokujoh E et al. [Randomized controlled study on the effectiveness of community pharmacists' advice for smoking cessation by Nicorette--evaluation at three months after initiation]. [Japanese]. *Yakugaku Zasshi.* 2004;124:989-995. PMID 15577269.

Satou Y, Kanda J, Okumura M, Nishida K. [An analysis of the educational effects of group counseling with visual aids: efforts to prevent diabetes in a business office setting]. [Japanese]. *Sangyo Eiseigaku Zasshi.* 2004;46:117-121. PMID 15382712.

Sawada A, Sakata N, Higuchi B et al. [Comparison of micafungin and fosfluconazole as prophylaxis for invasive fungal infection during neutropenia in children undergoing chemotherapy and hematopoietic stem cell transplantation]. [Japanese]. *Rinsho Ketsueki.* 2009;50:1692-1699. PMID 20068276.

Sekine Y, Takai Y, Nishii O et al. [Establishment of an optimum bowel preparation method before gynecologic laparoscopic surgery]. [Japanese]. *Yakugaku Zasshi.* 2001;121:637-645. PMID 11523124.

Sugiura M, Hata Y, Fukuda T et al. [One-week application of terbinafine cream compared with four-week application in treatment of Tinea pedis]. [Japanese]. *Nihon Ishinkin Gakkai Zasshi.* 2001;42:223-228. PMID 11704752.

Takahashi M, Araki A, Ito H. [Development of a new method for simple dietary education in elderly individuals with diabetes mellitus]. [Japanese]. *Nihon Ronen Igakkai Zasshi.* 2002;39:527-532. PMID 12404749.

### Spanish

Bonetto G, Salvatico E, Varela N, Cometto C, Gomez PF, Calvo B. [Pain prevention in term neonates: randomized trial for three methods]. [Spanish].  *Arch Argent Pediatr.* 2008;106:392-396. PMID 19030637.

Ceriani Cernadas JM, Carroli G, Pellegrini L et al. [The effect of early and delayed umbilical cord clamping on ferritin levels in term infants at six months of life: a randomized, controlled trial]. [Spanish]. *Arch Argent Pediatr.* 2010;108:201-208. PMID 20544134.

de Luis DA, de la FB, Izaola O et al. [Randomized clinical trial with a inulin enriched cookie on risk cardiovascular factor in obese patients]. [Spanish]. *Nutr Hosp.* 2010;25:53-59. PMID 20204256.

Garcia-Talavera Espin NV, Gomez Sanchez MB, Zomeno Ros AI et al. [Comparative study of two enteral feeding formulas in hospitalized elders: casein versus soybean protein]. [Spanish]. *Nutr Hosp.* 2010;25:606-612. PMID 20694297.

Gomez-Garcia A, Hernandez-Salazar E, Gonzalez-Ortiz M, Martinez-Abundis E. [Effect of oral zinc administration on insulin sensitivity, leptin and androgens in obese males]. [Spanish]. *Rev Med Chil.* 2006;134:279-284. PMID 16676098.

Lopez-De-Blanc SA, Salati-De-Mugnolo N, Femopase FL et al. Antifungal topical therapy in oral chronic candidosis. A comparative study. *Med Oral.* 2002;7:260-270. PMID 12134127.

Martinez Gonzalez JM, Benito PB, Fernandez CF, San Hipolito ML, Penarrocha DM. A comparative study of direct mandibular nerve block and the Akinosi technique. *Med Oral.* 2003;8:143-149. PMID 12618675.

Perez-Barcena J, Barcelo B, Homar J et al. [Comparison of the effectiveness of pentobarbital and thiopental in patients with refractory intracranial hypertension. Preliminary report of 20 patients]. [Spanish]. *Neurocirugia (Astur ).* 2005;16:5-12. PMID 15756405.

Rodriguez MC, Castano SC, Garcia OL, Recio Rodriguez JI, Castano SY, Gomez Marcos MA. [Efficacy of an educational intervention group on changes in lifestyles in hypertensive patients in primary care: a randomized clinical trial]. [Spanish]. *Rev Esp Salud Publica.* 2009;83:441-452. PMID 19701575.

Vasquez AM, Sanin F, Alvarez LG, Tobon A, Rios A, Blair S. [Therapeutic efficacy of a regimen of artesunate-mefloquine-primaquine treatment for Plasmodium falciparum malaria and treatment effects on gametocytic development]. [Spanish]. *Biomedica.* 2009;29:307-319. PMID 20128355.

### English

Davidson JA, Einhorn D, Allweiss P et al. Effect of premixed nph and regular insulin on glucose control and health-related quality of life in patients with type 2 diabetes mellitus. *Endocr Pract.* 1997;3:331-336. PMID 15251769.

Friedman Z, Katznelson R, Phillips SR et al. A randomized double-blind comparison of a morphine-fentanyl combination vs. morphine alone for patient-controlled analgesia following bowel surgery. *Pain Pract.* 2008;8:248-252. PMID 18503621.

Fu S, Choy NL, Nitz J. Controlling balance decline across the menopause using a balance-strategy training program: a randomized, controlled trial. *Climacteric.* 2009;12:165-176. PMID 19058060.

Halkes PH, van GJ, Kappelle LJ, Koudstaal PJ, Algra A. Medium intensity oral anticoagulants versus aspirin after cerebral ischaemia of arterial origin (ESPRIT): a randomised controlled trial. *Lancet Neurol.* 2007;6:115-124. PMID 17239798.

Ize-Iyamu IN, Saheeb BD. Feeding intervention in cleft lip and palate babies: a practical approach to feeding efficiency and weight gain. *Int J Oral Maxillofac Surg.* 2011;40:916-919. PMID 21641186.

Lyytinen J, Kaakkola S, Gordin A, Kultalahti ER, Teravainen H, Sovijarvi A. The effect of COMT inhibition with entacapone on cardiorespiratory responses to exercise in patients with Parkinson's disease. *Parkinsonism Relat Disord.* 2002;8:349-355. PMID 15177064.

Malpuech-Brugere C, Mouriot J, Boue-Vaysse C et al. Differential impact of milk fatty acid profiles on cardiovascular risk biomarkers in healthy men and women. *Eur J Clin Nutr.* 2010;64:752-759. PMID 20485306.

Odergren A, Algvere PV, Seregard S, Libert C, Kvanta A. Vision-related function after low-dose transpupillary thermotherapy versus photodynamic therapy for neovascular age-related macular degeneration. *Acta Ophthalmol.* 2010;88:426-430. PMID 20597872.

Solon FS, Sarol JN, Jr., Bernardo AB et al. Effect of a multiple-micronutrient-fortified fruit powder beverage on the nutrition status, physical fitness, and cognitive performance of schoolchildren in the Philippines. *Food Nutr Bull.* 2003;24:S129-S140. PMID 17016955.

Wang WC, Morales KH, Scher CD et al. Effect of long-term transfusion on growth in children with sickle cell anemia: results of the STOP trial. *J Pediatr.* 2005;147:244-247. PMID 16126058.
