# Supplementary material for: Data extraction from machine-translated versus original language randomized trial reports: a comparative study
Source: Syst Rev. 2013 Nov 7;2:97. doi: 10.1186/2046-4053-2-97 (PMC4226266; doi:10.1186/2046-4053-2-97)
Supplement: Additional file 2: Table S1 — Characteristics of included trials. Table S2: Translation time (minutes), by language. Table S3: Estimated additional time required compared to extraction of a similar English article. Table S4: Confidence in accuracy and completeness of the translation. [file 2046-4053-2-97-S2.docx]

Additional file 2. Supplemental tables

Table S1. Characteristics of included trials

| **Language** | **Publication Dates (N)** | **Clinical Domains / Populations** | **Intervention Types (N)** | **Outcome Types (N)** |
| --- | --- | --- | --- | --- |
| Chinese (simplified) | 2004 (1)  2008 (3)  2009 (1)  2010 (4)  2011 (1) | Colonoscopy  Dementia  Gynecologic surgery  Hepatocellular carcinoma  Lung cancer  Myopia  Nocturia  Parkinson’s Disease  Premature LBW infants  Pulmonary surgery | Behavior (1)  CAM (4)  Diagnostic test agent (1)  Drug (4) | Dichotomous (8)  Continuous (9) |
| French | 1993 (1)  1994 (1)  1996 (1)  1997 (1)  2000 (1)  2006 (2)  2008 (2)  2009 (1) | Acute sinusitis  Allergic conjunctivitis  Helicobacter pylori infection  Hepatitis C  Homeopathy adverse effect  Hypercholesterolemia  Hypertension  Obstetrics  Pterygium (ophthalmology)  Refraction (ophthalmology) | Counseling (1)  CAM (1)  Diet (1)  Drug (7) | Dichotomous (7)  Continuous (8) |
| German | 2002 (1)  2005 (1)  2007 (2)  2008 (2)  2009 (1)  2010 (2)  2012 (1) | Anesthesia  Cataract surgery  Chronic prostatitis  Hernia surgery  Keratoplasty (ophthalmology)  Knee arthroscopic surgery  Scar formation  Schizophrenia  Total hip replacement  Whiplash | CAM (1)  Counseling (1)  Device (3)  Drug (3)  Exercise (1)  Surgery (1) | Dichotomous (7)  Continuous (10) |
| Japanese | 2001 (2)  2002 (1)  2004 (2)  2008 (1)  2009 (2)  2010 (2) | Cardiac function  Colorectal cancer  Diabetes mellitus education  Diabetes mellitus prevention  Fungal prophylaxis (oncology), 2 studies  Gynecologic laparoscopic surgery  Hypercholesterolemia  Smoking cessation  Tinea pedis | CAM (1)  Counseling (1)  Drug (4)  Education (3)  Formulation (1) | Dichotomous (5)  Continuous (7) |
| Spanish | 2002 (1)  2003 (1)  2005 (1)  2006 (1)  2008 (1)  2009 (2)  2010 (3) | Gerontology, enteral feedings  Hypertension  Intracranial hypertension  Malaria  Molar extraction (dental)  Neonatology, 2 studies  Obesity, 2 studies  Oral candidiasis | Anesthesia (1)  Drug (5)  Education (1)  Nutrition (2)  Procedure (1) | Dichotomous (5)  Continuous (7) |
| English | 1997 (1)  2002 (1)  2003 (1)  2005 (1)  2007 (1)  2008 (1)  2009 (1)  2010 (2)  2011 (1) | Anesthesia, bowel surgery  Cardiovascular risk factors  Cerebral ischemia  Cleft lip and palate  Diabetes mellitus, type 2  Macular degeneration  Menopause  Nutrition, micronutrients  Parkinson’s disease  Sickle cell anemia | Blood products (1)  Drugs (4)  Exercise (1)  Nutrition (3)  Procedure (1) | Dichotomous (6)  Continuous (9) |

CAM = complementary and alternative medicine; LBW = low birth weight; N = number of articles.

Table S2. Translation time (minutes), by language

| **Articles*:** | **1** | **2** | **3** | **4** | **5** | **6** | **7** | **8** | **9** | **10** | **Median** |
| --- | --- | --- | --- | --- | --- | --- | --- | --- | --- | --- | --- |
| **European** |  |  |  |  |  |  |  |  |  |  |  |
| French | 20 | 30 | 30 | 30 | 30 | 30 | 30 | 30 | 45 | 60 | 30 |
| German | 15 | 20 | 20 | 25 | 30 | 30 | 30 | 30 | 40 | 240 | 30 |
| Spanish | 10 | 10 | 10 | 10 | 15 | 15 | 15 | 15 | 20 | 20 | 15 |
| **Asian** |  |  |  |  |  |  |  |  |  |  |  |
| Chinese | 60 | 60 | 60 | 60 | 60 | 60 | 60 | 60 | 60 | 120 | 60 |
| Japanese | 5 | 5 | 20 | 20 | 20 | 20 | 30 | 30 | 30 | 60 | 20 |

* For each language, the approximate duration of time, in minutes, for translation of each article is listed, sorted from shortest to longest time.

Table S3. Estimated additional time required compared with extraction of a similar English article

| Extra Time | Chinese  Percent (n) | French  Percent (n) | German  Percent (n) | Japanese  Percent (n) | Spanish  Percent (n) | Overall  Percent (n) |
| --- | --- | --- | --- | --- | --- | --- |
| <5 min | 20% (4) | 5% (1) | 20% (4) | 5% (1) | 56% (10) | 21% (20) |
| 6-30 min | 70% (14) | 68% (13) | 60% (12) | 75% (15) | 39% (7) | 63% (61) |
| >30 min | 10% (2) | 26% (5) | 20% (4) | 20% (4) | 6% (1) | 16% (16) |

**Table S4. Confidence in accuracy and completeness of the translation**

| Confidence | Chinese  Percent (n) | French*  Percent (n) | German  Percent (n) | Japanese  Percent (n) | Spanish  Percent (n) | Overall*  Percent (n) |
| --- | --- | --- | --- | --- | --- | --- |
| Strong | 10% (2) | 5% (1) | 15% (3) | 5% (1) | 60% (12) | 26% (26) |
| Moderate | 65% (13) | 65% (13) | 65% (13) | 60% (12) | 25% (5) | 55% (55) |
| Little | 25% (5) | 25% (5) | 20% (4) | 35% (7) | 15% (3) | 18% (18) |

* 1 extractor did not rate confidence level for 1 article.
