# Supplementary material for: Data extraction from machine-translated versus original language randomized trial reports: a comparative study
Source: Syst Rev. 2013 Nov 7;2:97. doi: 10.1186/2046-4053-2-97 (PMC4226266; doi:10.1186/2046-4053-2-97)
Supplement: Additional file 3 — Summary description of data extractors. [file 2046-4053-2-97-S3.docx]

Additional file 3. Summary description of data extractors

Among the 15 investigators who extracted data, 11 are MD- or PhD- (or both) researchers, two are research associates (or equivalent), and two are medical residents with research experience. The median duration of experience with data extraction was 5 years, with four extractors having 10 to 14 years of experience, and four having less than 1 year of experience. Six of the investigators have participated in more than 20 systematic reviews, one has participated in 11 to 20 reviews, five in 6 to 10 reviews, and three in 5 or fewer reviews. Eight investigators have extracted more than 100 studies, four have extracted 51 to 100 studies, and three have extracted 50 or fewer articles. Nine investigators judged that they have a lot of comfort with the Cochrane risk of bias questions, four had moderate comfort, one had little comfort, and one had no experience with assessing risk of bias. The medical resident with no prior systematic review experience extracted 10 original language articles. She had oversight and assistance from the director of the EPC she was affiliated with.
