# Supplementary material for: Data extraction from machine-translated versus original language randomized trial reports: a comparative study
Source: Syst Rev. 2013 Nov 7;2:97. doi: 10.1186/2046-4053-2-97 (PMC4226266; doi:10.1186/2046-4053-2-97)
Supplement: Additional file 4 — Flowchart of basic processes. [file 2046-4053-2-97-S4.docx]

Additional file 4. Flowchart of basic processes
