# Supplementary material for: Data extraction from machine-translated versus original language randomized trial reports: a comparative study
Source: Syst Rev. 2013 Nov 7;2:97. doi: 10.1186/2046-4053-2-97 (PMC4226266; doi:10.1186/2046-4053-2-97)
Supplement: Additional file 5 — Data extraction items. [file 2046-4053-2-97-S5.docx]

**Additional file 5. Data extraction items**

| **Domain** | **Extraction Item** | **Entry type** | **Options** | **Analyzed?** |
| --- | --- | --- | --- | --- |
| Design | Inclusion criteria | Free text | -- | Yes |
| Design | Exclusion criteria | Free text | -- | Yes |
| Design | Funding source | Check boxes with free text option (multiple choice allowed) | - Government - Industry - Academic or Hospital - Foundation etc. - Named entity of an unclear category (free text for name) - No funding (explicitly stated) - Not reported | Yes |
| Design | No. centers | Radio buttons with free text option (single answer allowed) | - Single center - Multicenter (how many centers? - Not reported/unclear | Yes |
| Design | Followup duration (maximum or mean) | Free text | -- | Yes |
| Design | Randomization technique reported? | Radio buttons | - Yes - No | Yes |
| Design | Allocation concealment method reported? | Radio buttons | - Yes - No | Yes |
| Design | Intention-to-treat analysis reported for any outcome (either in methods or results sections) | Radio buttons | - Yes - No | Yes |
| Design | Power calculation reported for any outcome | Radio buttons | - Yes - No | Yes |
| Design | Subject blinding explicitly reported (for any outcome) | Radio buttons | - Yes - No | Yes |
| Design | Caregiver blinding explicitly reported (for any outcome) | Radio buttons | - Yes - No | Yes |
| Design | Outcome assessor blinding explicitly reported (for any outcome) | Radio buttons | - Yes - No | Yes |
| Design | Double blinded(for any outcome) | Radio buttons | - Yes - No | Yes |
| Design | Single blinded(for any outcome) | Radio buttons | - Yes - No | Yes |
| Design | Select all outcomes reported from a given list of about 12 outcomes | Check boxes (multiple choices allowed) | - Different outcomes for each language culled from the articles - None of these outcomes found | Yes |
| Intervention / Comparator | Study arm title | Free text | -- | No |
| Intervention / Comparator | Study arm description | Free text | -- | No (inadequate nonredundant data were extracted) |
| Intervention / Comparator | Dose (individually for each outcome) | Free text | -- | Yes (for 1 or 2 preselected outcomes only) |
| Intervention / Comparator | Frequency (individually for each outcome) | Free text | -- | Yes (for 1 or 2 preselected outcomes only) |
| Intervention / Comparator | Route (individually for each outcome) | Free text | -- | Yes (for 1 or 2 preselected outcomes only) |
| Intervention / Comparator | Duration (individually for each outcome) | Free text | -- | Yes (for 1 or 2 preselected outcomes only) |
| Intervention / Comparator | No. randomized into each study arm (for all interventions) | Free text | -- | Yes (for 1 or 2 preselected outcomes only) |
| Outcome | Outcome title | Free text | -- | No (1 preselected continuous and/or 1 preselected categorical outcome) |
| Outcome | Outcome units | Free text | -- | No (inadequate meaningful data were extracted) |
| Outcome | Outcome description | Free text | -- | Yes |
| Results | Continuous: No. analyzed (per intervention) | Free text | -- | Yes |
| Results | Continuous: Mean or median reported†† | Free text | “Mean” or “Median” (not the value) | Yes |
| Results | Continuous: Net difference (or difference between final) | Free text | (calculate if necessary) | Yes |
| Results | Continuous: Standard error of net difference | Free text | (calculate if necessary) | Yes |
| Results | Categorical: No. analyzed (per intervention) | Free text | -- | Yes |
| Results | Categorical: No. events (counts) | Free text | -- | Yes (combined with reported odds ratio and confidence interval) |
| Results | Categorical: Odds ratio and 95% confidence interval | Free text | (calculate if necessary) | Only if there were no counts data (only reported data; calculations not analyzed) |
| Results | Both: Reported P value of difference or odds ratio | Free text | -- | Yes |
| Results | Both: What analysis adjusted for | Free text | -- | No (insufficient data) |
| Study Quality | What is the risk of selection bias (biased allocation to interventions) due to inadequate generation of a randomized sequence? | Pull down menu | - Low - Unclear - High | Yes |
| Study Quality | What is the risk of selection bias (biased allocation to interventions) due to inadequate concealment of allocations before assignment? | Pull down menu | - Low - Unclear - High | Yes |
| Study Quality | For the preselected categorical outcome (or continuous outcome if there is no categorical outcome), what is the risk of performance bias due to knowledge of the allocated interventions by participants and personnel during the study (lack of study participant and personnel blinding)? | Pull down menu | - Low - Unclear - High | Yes |
| Study Quality | For the preselected categorical outcome (or continuous outcome if there is no categorical outcome), what is the risk of detection bias due to knowledge of the allocated interventions by outcome assessment (lack of outcome assessor blinding)? | Pull down menu | - Low - Unclear - High | Yes |
| Study Quality | For the preselected categorical outcome (or continuous outcome if there is no categorical outcome), what is the risk of attrition bias due to amount, nature, or handling of incomplete outcome data? | Pull down menu | - Low - Unclear - High | Yes |
| Miscellaneous | For TRANSLATED articles: How much additional time do you estimate the extraction of the translated article took compared to an extraction of a similar English article? | Free text | - <5 minutes extra time - 6-30 min extra time - >30 min extra time | Yes |
| Miscellaneous | For TRANSLATED articles: How confident are you in the accuracy and completeness of the translation of the original article? | Free text | - I have little confidence - I have a moderate confidence * - I have strong confidence | Yes |
| Miscellaneous | For TRANSLATED articles: Please provide examples of poor translations that made extraction difficult | Free text | -- | C |
