# Supplementary material for: Data extraction from machine-translated versus original language randomized trial reports: a comparative study
Source: Syst Rev. 2013 Nov 7;2:97. doi: 10.1186/2046-4053-2-97 (PMC4226266; doi:10.1186/2046-4053-2-97)
Supplement: Additional file 6 — Percentage of correct extractions, per item and language, adjusted for individual’s likelihood of correctly extracting the same data item from English articles. [file 2046-4053-2-97-S6.docx]

Additional file 6. Percentage of correct extractions, per item and language, adjusted for individual’s likelihood of correctly extracting the same data item from English articles

| **Domain** | **Extraction Item** | **English** | **Chinese** | **French** | **German** | **Japanese** | **Spanish** |
| --- | --- | --- | --- | --- | --- | --- | --- |
| Intervention | Route (of all interventions) | 100 | 99 | 100 | 100 | 100 | 100 |
| Results | No. analyzed (per intervention) | 100 | 96 | 98 | 98 | 94 | 98 |
| Design | Inclusion criteria | 99 | 99 | 100 | 98 | 93 | 99 |
| Design | Power calculation | 99 | 95 | 74 | 97 | 100 | 98 |
| Design | Single blinded* | 99 | 100 | 95 | 100 | 90 | 100 |
| Results | No. events (counts) or odds ratio † | 99 | 98 | 86 | 37 | 47 | 94 |
| Design | Exclusion criteria | 98 | 97 | 100 | 70 | 79 | 97 |
| Design | Intention-to-treat analysis* | 98 | 100 | 90 | 95 | 80 | 75 |
| Design | No extra outcomes added ‡ | 96 | 90 | 82 | 99 | 96 | 99 |
| Intervention | Duration (of all interventions) | 96 | 28 | 75 | 66 | 88 | 77 |
| Design | Randomization technique | 95 | 87 | 82 | 78 | 89 | 93 |
| Design | Double blinded* | 94 | 100 | 100 | 95 | 100 | 95 |
| Results | Mean or median reported*^,^§ | 94 | 100 | 100 | 84 | 71 | 100 |
| Design | Funding source | 93 | 100 | 86 | 99 | 99 | 35 |
| Design | Allocation concealment method* | 93 | 100 | 85 | 100 | 80 | 85 |
| Design | Subject blinding (explicit) | 93 | 100 | 98 | 97 | 90 | 78 |
| Results | Reported P value of difference or odds ratio | 93 | 30 | 81 | 73 | 21 | 73 |
| Design | Caregiver blinding (explicit) | 92 | 100 | 95 | 96 | 98 | 98 |
| Design | Outcome assessor blinding (explicit) | 89 | 88 | 91 | 97 | 96 | 90 |
| Design | Followup duration | 88 | 79 | 41 | 82 | 84 | 96 |
| Intervention | Frequency (of all interventions) | 87 | 74 | 87 | 86 | 75 | 83 |
| Outcome | Description | 87 | 45 | 51 | 42 | 75 | 98 |
| Design | No. centers | 86 | 97 | 85 | 87 | 51 | 87 |
| Intervention | Dose (of all interventions) | 82 | 88 | 97 | 79 | 91 | 81 |
| Results | Net difference § | 81 | 28 | 15 | 73 | 67 | 60 |
| Results | Standard error of net difference § | 81 | 38 | 15 | 64 | 56 | 71 |
| Design | No outcomes missed \|\| | 75 | 80 | 74 | 13 | 11 | 31 |
| Intervention | No. randomized (for all interventions) | 71 | 97 | 91 | 97 | 82 | 89 |
| Design | Outcomes reported ¶ | 63 | 36 | 43 | 12 | 5 | 21 |
|  |  |  |  |  |  |  |  |
| Overall** | % items that are each ≥98% correct | 30 | 41 | 26 | 22 | 19 | 30 |
| Overall | % items that are each ≥91% correct | 63 | 59 | 37 | 48 | 30 | 48 |
| Overall | % items that are each ≥76% correct | 93 | 74 | 74 | 70 | 67 | 78 |
| Overall | % items that are each ≥51% correct | 100 | 78 | 85 | 89 | 89 | 93 |
| Overall | % items that are each ≤50% correct | 0 | 22 | 15 | 11 | 11 | 7 |

Extraction items are sorted by accuracy in English. Shading of cells matches reported percentages. Darker shading indicates greater inaccuracy:

| 98-100% correct  (100^th^ percentile) | 91-97% correct  (72^nd^ percentile) | 76-90% correct  (50^th^ percentile) | 51-75% correct  (24^th^ percentile) | ≤50% correct  (11^th^ percentile). |
| --- | --- | --- | --- | --- |

* Crude (unadjusted) percentage.

† For dichotomous outcomes.

‡ No outcomes not found in the original article were added from the translated article. This item is excluded from the overall percentages of items correct at the bottom of the table.

§ For continuous outcomes.

|| No outcomes found in the original article were missed from the translated article. This item is excluded from the overall percentages of items correct at the bottom of the table.

¶ From a list of proffered outcomes, there was exact agreement as to which were reported in the study.

** The five “Overall” rows display the percentage of the 29 items (not the individual extractions), per language, that were each extracted correctly the given percentage of the time (e.g., 30% of the English items were extracted correctly 98-100% of the time). Note that the final two rows, by definition, sum to 100%.
