# Supplementary material for: Data extraction from machine-translated versus original language randomized trial reports: a comparative study
Source: Syst Rev. 2013 Nov 7;2:97. doi: 10.1186/2046-4053-2-97 (PMC4226266; doi:10.1186/2046-4053-2-97)
Supplement: Additional file 7 — Odds ratios of correct extractions, compared with English, adjusted for individual’s likelihood of correctly extracting the same data item from English articles. The extraction items are sorted to match Additional file 6. Shading of cells matches is based on the odds ratio and statistical significance. Darker shading indicates greater inaccuracy; NS = non-significant; OR = odds ratio. [file 2046-4053-2-97-S7.docx]

Additional file 7. Odds ratios (95% confidence intervals) of correct extractions, compared with English, adjusted for individual’s likelihood of correctly extracting the same data item from English articles

| **Domain** | **Extraction Item** | **Chinese** | **French** | **German** | **Japanese** | **Spanish** |
| --- | --- | --- | --- | --- | --- | --- |
| Intervention | Route (of all interventions) | <0.1 (<0.1, 2.9) | 0.3 (<0.1, >5) | 0.2 (<0.1, >5) | 0.5 (<0.1, >5) | >5 (<0.1, >5) |
| Results | No. analyzed (per intervention) | <0.1 (<0.1, 0.4) | <0.1 (<0.1, 0.9) | <0.1 (<0.1, 0.9) | <0.1 (<0.1, 0.3) | <0.1 (<0.1, 1.3) |
| Design | Inclusion criteria | 1.9 (0.1, >5) | 3.0 (0.2, >5) | 0.5 (<0.1, >5) | 0.1 (<0.1, 1.4) | 0.8 (0.1, >5) |
| Design | Power calculation | 0.3 (<0.1, 3.9) | <0.1 (<0.1, 0.7) | 0.4 (<0.1, >5) | >5 (<0.1, >5) | 0.6 (<0.1, >5) |
| Design | Single blinded* | >5 (<0.1, >5)* | 1.9 (<0.1, 4)* | 1.2 (<0.1, >0.5)* | 0.8 (<0.1, 1.3)* | 0.6 (<0.1, >5)* |
| Results | No. events (counts) or odds ratio † | 0.8 (<0.1, >5) | <0.1 (<0.1, 1.9) | <0.1 (<0.1, 0.3) | <0.1 (<0.1, 0.5) | 0.2 (<0.1, >5) |
| Design | Exclusion criteria | 0.6 (<0.1, >5) | >5 (<0.1, >5) | <0.1 (<0.1, 0.7) | <0.1 (<0.1, 1.2) | 0.7 (<0.1, >5) |
| Design | Intention-to-treat analysis* | 1.3 (0.1, >5)* | 0.2 (<0.1, 1.7)* | 0.5 (<0.1, >5)* | 0.1 (<0.1, 0.6)* | 0.1 (<0.1, 0.4)* |
| Design | No extra outcomes added ‡ | 0.4 (<0.1, >5) | 0.2 (<0.1, 4.8) | 3.0 (0.1, 5) | 1.1 (<0.1, >5) | 4.1 (0.1, >5) |
| Intervention | Duration (of all interventions) | <0.1 (<0.1, 0.2) | 0.1 (<0.1, 0.9) | <0.1 (<0.1, 0.7) | 0.3 (<0.1, 2.3) | 0.1 (<0.1, 0.9) |
| Design | Randomization technique | 0.3 (<0.1, 2.2) | 0.2 (<0.1, 1.2) | 0.2 (<0.1, 1.0) | 0.4 (0.1, 2.8) | 0.7 (0.1, >5) |
| Design | Double blinded* | 2.4 (0.2, >5)* | 0.1 (0.2, >5)* | 0.5 (0.1, >5)* | 2.4 (0.2, >5)* | 1.0 (0.1, >5)* |
| Results | Mean or median reported*^,^§ | 2.8 (0.2, >5)* | 2.3 (0.1, >5)* | 0.4 (0.1, 1.6)* | 0.2 (<0.1, 0.7)* | 1.8 (0.1, >5)* |
| Design | Funding source | >5 (0.2, >5) | 0.5 (<0.1, >5) | >5 (0.2, >5) | >5 (0.2, >5) | <0.1 (<0.1, 1.0) |
| Design | Allocation concealment method* | 3.6 (0.2, >5)* | 0.5 (0.1, 2.0)* | 3.6 (0.2, >5)* | 0.3 (0.1, 1.3)* | 0.5 (0.1, 2.0)* |
| Design | Subject blinding (explicit) | >5 (<0.1, >5) | 4.4 (0.2, 104.1) | 2.5 (0.1, >5) | 0.6 (<0.1, >5) | 0.3 (<0.1, 4.1) |
| Results | Reported P value of difference or odds ratio | <0.1 (<0.1, 0.2) | 0.3 (0.1, 2.0) | 0.2 (<0.1, 1.1) | <0.1 (<0.1, 0.2) | 0.2 (<0.1, 1.3) |
| Design | Caregiver blinding (explicit) | >5 (<0.1, >5) | 1.7 (0.2, >5) | 2.0 (0.2, >5) | 4.3 (0.2, >5) | 3.3 (0.2, >5) |
| Design | Outcome assessor blinding (explicit) | 0.9 (0.2, >5) | 1.2 (0.3, >5) | 3.4 (0.4, >5) | 2.8 (0.3, >5) | 1.1 (0.2, >5) |
| Design | Followup duration | 0.5 (0.1, >5) | <0.1 (<0.1, 1.0) | 0.7 (0.1, >5) | 0.7 (0.1, >5) | 3.8 (0.3, >5) |
| Intervention | Frequency (of all interventions) | 0.4 (<0.1, 4.0) | 1.0 (0.1, >5) | 0.9 (0.1, >5) | 0.4 (0.1, 3.8) | 0.7 (0.1, >5) |
| Outcome | Description | 0.1 (<0.1, 0.8) | 0.2 (<0.1, 1.5) | 0.1 (<0.1, 1.0) | 0.5 (0.1, 2.8) | >5 (0.6, >5) |
| Design | No. centers | 4.7 (0.4, >5) | 0.9 (0.2, >5) | 1.1 (0.2, >5) | 0.2 (<0.1, 0.9) | 1.1 (0.2, >5) |
| Intervention | Dose (of all interventions) | 1.6 (0.4, >5) | >5 (0.9, >5) | 0.8 (0.2, 2.8) | 2.1 (0.4, >5) | 0.9 (0.2, 4.2) |
| Results | Net difference § | <0.1 (<0.1, 0.7) | <0.1 (<0.1, 0.4) | 0.6 (0.1, 3.7) | 0.5 (0.1, 3.6) | 0.3 (<0.1, 3.2) |
| Results | Standard error of net difference § | 0.1 (<0.1, 0.9) | <0.1 (<0.1, 0.5) | 0.4 (0.1, 2.6) | 0.3 (<0.1, 2.3) | 0.6 (0.1, >5) |
| Design | No outcomes missed \|\| | 1.3 (0.2, >5) | 0.9 (0.2, 4.7) | <0.1 (<0.1, 0.3) | <0.1 (<0.1, 0.4) | 0.1 (<0.1, 0.8) |
| Intervention | No. randomized (for all interventions) | >5 (1.1, >5) | 4.3 (0.6, >5) | >5 (1.1, >5) | 1.8 (0.3, >5) | 3.2 (0.5, >5) |
| Design | Outcomes reported ¶ | 0.3 (0.1, 1.8) | 0.4 (0.1, 2.5) | <0.1 (<0.1, 0.5) | <0.1 (<0.1, 0.4) | 0.2 (<0.1, 1.0) |

The extraction items are sorted to match Additional file 6. Shading of cells matches is based on the odds ratio and statistical significance. Darker shading indicates greater inaccuracy:

| OR≥1 | 0.5<OR<1 (NS) | 0.1<OR≤0.5 (NS) | OR≤0.1 (NS) | Statistically significant (OR<0.1) |
| --- | --- | --- | --- | --- |

NS = non-significant; OR = odds ratio.

* Odds ratio based on crude (unadjusted) proportions correct.

† For dichotomous outcomes.

‡ No outcomes not found in the original article were added from the translated article.

§ For continuous outcomes.

|| No outcomes found in the original article were missed from the translated article.

¶ From a list of proffered outcomes, there was exact agreement as to which were reported in the study.
